# transFusion: a novel comprehensive platform for integration analysis of single-cell and spatial transcriptomics

**DOI:** 10.1093/bioinformatics/btag059

**Published:** 2026-02-05

**Authors:** Weiqiang Lin, Xinyi Xiao, Chuan Qiu, Hui Shen, Hongwen Deng

**Affiliations:** Tulane Center for Biomedical Informatics and Genomics, Deming Department of Medicine, School of Medicine, Tulane University, New Orleans, LA 70112, United States; Department of Epidemiology, School of Public Health and Tropical Medicine, Tulane University, New Orleans, LA 70112, United States; Tulane Center for Biomedical Informatics and Genomics, Deming Department of Medicine, School of Medicine, Tulane University, New Orleans, LA 70112, United States; Tulane Center for Biomedical Informatics and Genomics, Deming Department of Medicine, School of Medicine, Tulane University, New Orleans, LA 70112, United States; Tulane Center for Biomedical Informatics and Genomics, Deming Department of Medicine, School of Medicine, Tulane University, New Orleans, LA 70112, United States

## Abstract

**Motivation:**

Understanding spatial organization, intercellular interactions, and regulatory networks within the spatial context of tissues is crucial for uncovering complex biological processes and disease mechanisms. Spatial transcriptomics technologies have revolutionized this field by enabling the spatially resolved profiling of gene expression. 10× Visium has emerged as the predominant spatial technology, but its low resolution and the complexity of integrating multimodal datasets present significant analytical challenges, particularly for researchers with limited computational and statistical expertise. Current spatial transcriptomics analysis platforms generally fall short of effectively integrating multimodal data and maximizing the utility of spatial information—such as uncovering complex cellular spatial dependencies, multimodal gradient patterns, and spatial coexpression of ligand–receptor pairs and regulatory networks related to disease or biological states—thereby limiting their ability to provide comprehensive end-to-end analytical workflows when analyzing 10× Visium data.

**Results:**

To address these limitations, we developed transFusion, a novel, advanced web-based platform specializing in the most comprehensive and effective integration analysis of scRNA-seq and 10× Visium spatial transcriptomics data. transFusion offers 12 key functions, from basic visualization to advanced analyses, including intercellular dependency analysis, ligand–receptor coexpression identification and visualization, and spatial multimodal gradient variation patterns. Two case studies were used to demonstrate transFusion’s capabilities in exploring tissue architecture, intercellular communication, dependency networks, and multimodal gradient variation patterns with minimal computational skills and statistical expertise. transFusion provides a flexible and powerful framework for multimodal data integration analysis.

**Availability and implementation:**

transFusion is freely available at https://github.com/WQLin8/transFusion.

## 1 Introduction

Recent advances in high-throughput sequencing technologies have revolutionized our understanding of tissue complexity at unprecedented molecular resolutions. Single-cell RNA sequencing (scRNA-seq) has emerged as a powerful tool for identifying cellular subtypes, revealing distinct cellular compositions and states (Regev *et al.* 2017, Stubbington *et al.* 2017, Birnbaum 2018, Tokura *et al.* 2022). However, the spatial organization of cells within tissues plays a fundamental role in shaping their function, interaction networks, and contributions to tissue homeostasis, which are often lost in dissociation-based single-cell techniques (Lähnemann *et al.* 2020). Advances in spatial transcriptomics (ST), such as 10× Visium (Ståhl *et al.* 2016), Slide-seq (Rodriques *et al.* 2019), and Stereo-seq (Chen *et al.* 2022), allow for gene expression profiling while preserving spatial context, offering a more complete picture of tissue biology. The 10× Visium has been recognized as the most popular sequencing-based method. A wealth of valuable ST data has been generated based on 10× Visium (Robles-Remacho *et al.* 2023). However, with a spatial resolution of 55 µm, 10× Visium is unable to achieve single-cell resolution, making the integration of scRNA-seq data essential for comprehensive multimodal tissue analysis. This powerful combination of ST and scRNA-seq data has been widely applied across numerous research domains to deepen our understanding of the complex microenvironments of tissues (Fang *et al.* 2023, Zhang, *et al.* 2024, Lin *et al.* 2025).

Meanwhile, a wide array of computational tools has been developed to handle various aspects of spatial and single-cell transcriptomics data analysis. These tools support tasks such as performing cell-type deconvolution for spot-resolution ST (Cable *et al.* 2022, Kleshchevnikov *et al.* 2022, Ma and Zhou 2022), identifying differentially expressed genes in specific cell types or spatial regions/clusters (Stuart *et al.* 2019), inferring intercellular interaction patterns (Efremova *et al.* 2020, Jin *et al.* 2025) and ligand–receptor coexpression pattern (Liu *et al.* 2022) and disease-related regulatory networks (Morabito *et al.* 2023). While these methods have greatly expanded the biological questions that can be addressed, the abundance and specificity of these tools present significant challenges to the research community. Many of these methods require substantial computational programming skills and statistical expertise, creating a formidable barrier for biologists without extensive statistical expertise and computational programming skills. As a result, a substantial volume of spatial transcriptomic data generated by 10× Visium remains underexplored, awaiting more comprehensive analysis and deeper insights.

Several ST analysis platforms have emerged, providing web-based solutions for visualizing and analyzing ST data. These platforms offer distinct advantages while all confront certain constraints. Cellar (Hasanaj *et al.* 2022), Stellaris (Li *et al.* 2023), WebSCST (Zhang *et al.* 2022), Aquila (Zheng *et al.* 2023), and spatialGE (Ospina *et al.* 2025) do not integrate scRNA-seq, which constrains the potential depth of biological insights that could be extracted from the analysis. Integrated platforms like STExplore (Wang *et al.* 2025), SRT-Server (Yang and Zhou 2024), and SpatialTME (Shi *et al.* 2024), despite proficiency in accommodating multiple sequencing technologies, fail to deliver a complete analytical framework tailored to the special needs of ST data generated by 10× Visium. The compatibility of cross-platforms compromises their ability to address the unique complexities associated with 10× Visium spatially resolved transcriptomic information and they lack ability to explore complex spatial cellular relationships and tissue architecture, for example, comprehensively studying cellular interdependency, spatial gradient variation pattern of gene expression, cellular compositions, and signaling pathways related to disease or biological states and complex regulatory networks. These limitations significantly constrain our ability to gain a comprehensive understanding of the biological processes and interactions that shape tissue organization and function.

Recognizing these limitations, we identify a pressing need for a more comprehensive and adaptable computational platform that can meet the evolving demands of biomedical researchers working with 10× Visium ST and scRNA-seq data. In this study, we developed transFusion, a comprehensive platform specializing in the integrated analysis of scRNA-seq and 10× Visium ST data. transFusion provides a standardized, nonconfiguration-required workflow with preoptimized parameters, eliminating parameter customization to ensure accessibility for users without statistical training while guaranteeing reproducible, reliable results. transFusion provides advanced capabilities that surpass currently available options (Table 1). We leveraged two datasets to showcase its ability to uncover the complicated tissue microenvironment. By addressing the fragmentation and accessibility issues in the current landscape of spatial and single-cell analysis tools, our work provides a powerful, user-friendly framework for comprehensive scRNA-seq and 10× Visium ST integration analysis. This platform has the potential to accelerate research across a wide range of biological contexts, from developmental biology to cancer research, by making sophisticated multimodal analyses accessible to a broader scientific community.

**Table 1:** Feature comparison of the web-based analytic platforms.

Analytic methods	transFusion	SpatialTME	Cellar	STExplore	SRT-Server	STellaris	webSCST	Aquila	spatialGE
Descriptive analysis	**✓**	**✓**	**✓**	**✓**		**✓**		**✓**	**✓**
Differential gene expression (DGE) analysis	**✓**	**✓**	**✓**	**✓**	**✓**			**✓**	**✓**
Gene set enrichment analysis (GSEA)	**✓**			**✓**			**✓**		**✓**
Gene Ontology (GO) enrichment analysis	**✓**	**✓**			**✓**				
Pathway activity enrichment analysis	**✓**								
Cell enrichment analysis	**✓**	**✓**					**✓**		
Cell–cell and cross-region communication analysis	**✓**	**✓**		**✓**	**✓**			**✓**	
Ligand–receptor colocalization analysis	**✓**					**✓**			
Deconvolution analysis (includes multiple popular deconvolution methods)	**✓**				**✓**				
Dependency analysis	**✓**								
spatialDistance analysis	**✓**								**✓**
High-dimensional weighted gene coexpression network analysis (hdWGCNA)	**✓**								

## 2 Materials and methods

### 2.1 Differential gene expression analysis

Differential gene expression (DGE) analysis between specified clusters or regions is conducted using Seurat (Stuart *et al.* 2019) (v4.3.0). The FindMarkers function is utilized for pairwise comparisons, while FindAllMarkers is applied to identify differentially expressed genes across all clusters. Genes are classified as significantly upregulated if they meet the criteria of an adjusted *P*-value (adj. *P*-value) ≤ .05 and an average log2 fold change (avg_log2FC) ≥ 0.1. Conversely, genes with an adj. *P*-value ≤ .05 and avg_log2FC ≤ −0.1 are defined as significantly downregulated.

### 2.2 Gene Ontology enrichment analysis

Differentially expressed genes are identified using the FindAllMarkers function in Seurat (v4.3.0), with genes having an adj. *P*-value≤.05 selected for subsequent pathway enrichment analysis. The enrichGO function from clusterProfiler (Wu *et al.* 2021) (v4.11.0.2) is then employed to detect enriched pathways across the identified niches or regions.

### 2.3 Gene set enrichment analysis

Gene set enrichment analysis (GSEA) is performed using the AddModuleScore function in Seurat (v4.3.0) to calculate module scores for the specified gene sets of interest. This approach generates an enrichment score for each spot based on the expression of genes within each spot, allowing us to assess the relative activity of predefined pathways or biological processes across different spots.

### 2.4 Pathway signaling activity analysis of scRNA-seq and ST data

PROGENy (Schubert *et al.* 2018, Holland *et al.* 2020) is a footprint-based approach that utilizes a comprehensive collection of publicly available signaling perturbation experiments to identify a core set of pathway-responsive genes in humans and mice. Pathway activity scores are computed using the progeny’s (v1.17.3) progeny function, incorporating the 500 most responsive genes per pathway. Average pathway activity scores are calculated to assess pathway activity across different cell types or niches. For each spatial transcriptomic spot, transFusion estimates signaling pathway activities by leveraging PROGENy’s model matrix and the mlm method from decoupleR (Badia-i-Mompel *et al.* 2022), utilizing the top 500 genes from each transcriptional footprint and SCTransform-normalized data.

### 2.5 Cell–cell communication analysis and ligand–receptor colocalization

To estimate ligand–receptor interactions between different cell types, transFusion integrates CellPhoneDB (Efremova *et al.* 2020) (v2) and CellChat (Jin *et al.* 2025) (v2) to infer ligand–receptor pairs from processed 10× Visium ST and scRNA-seq data. Significant cell–cell interactions are identified based on a *P*-value threshold of ≤.05. For ST data, ligand–receptor colocalization is assessed by evaluating the spatial connectivity of the network, which includes only connections between spots exhibiting high expression of both the ligand and the receptor. The spatial connectivity of this network is then quantified using the Earth Mover’s Distance, based on the degree distribution of the network (Liu *et al.* 2022).

### 2.6 Cell enrichment analysis

To assess cell-type enrichment within clusters or regions, transFusion employs multimodal intersection analysis (Moncada *et al.* 2020), which utilizes the hypergeometric cumulative distribution to evaluate the statistical significance of the overlap between cell type-specific and tissue region-specific gene sets. The background gene set for *P*-value calculation consists of the intersection of all genes present in both the ST and scRNA-seq count matrices. For each spatial spot, the AddModuleScore function is used to quantify cell-type enrichment level, leveraging a list of highly differentially expressed genes identified from scRNA-seq differential expression analysis. Genes are classified as highly differentially expressed if the difference in the percentage of cells expressing the gene between two groups exceeds 0.5.

### 2.7 Deconvolution analysis

transFusion incorporates 12 deconvolution methods, including SCDC (Dong *et al.* 2021), RCTD (Cable *et al.* 2022), MuSiC (Wang *et al.* 2019), DeconRNASeq (Gong and Szustakowski 2013), DestVI (Gong and Szustakowski 2013), DWLS (Tsoucas *et al.* 2019), SPOTlight (Elosua-Bayes *et al.* 2021), SpatialDWLS (Dong and Yuan 2021), Stereoscope (Andersson *et al.* 2020), cell2location (Kleshchevnikov *et al.* 2022), CARD (Ma and Zhou 2022), and STdeconvolve (Miller *et al.* 2022). To reduce server load, transFusion downsamples scRNA-seq data to 300 cells per cell type when performing deconvolution analysis.

### 2.8 Cell dependency analysis

Leveraging the multiview intercellular spatial modeling framework (Tanevski *et al.* 2022), transFusion assesses the importance of each principal cell type’s abundance to the presence of other dominant cell types within spatial transcriptomic spots. Cellular proportions, derived from cell deconvolution analysis, are adaptively integrated into this framework, producing a normalized significance score that reflects cell-type dependencies across spatial contexts, including colocalization or reciprocal inclusion. However, it is important to note that these inferred interactions do not imply causality. Additionally, to link tissue structures with tissue functions, transFusion applies the same modeling framework to analyze the spatial distribution of PROGENy pathway activities, using standardized scores to quantify pathway activities.

### 2.9 spatialDistance analysis

For spatialDistance analysis (Lin *et al.* 2025), transFusion provides a function that allows manual selection of spots covering the region of interest, guided by the Hematoxylin and Eosin (H&E) image as a reference. The Euclidean distances between the outermost spots of the selected region and all other spots are computed using scaled spatial coordinates. These distance metrics are then utilized to analyze gene expression patterns, cell-type proportions (estimated via deconvolution analysis), and signaling pathway activities relative to tissue depth within the tissue. To visualize and model spatial gradients of these molecular and cellular features, density-over-distance plots combined with LOESS regression are employed. By capturing these spatial dependencies, transFusion enhances the ability to interpret how cellular and molecular compositions vary across anatomical structures, offering deeper insights into spatially organized biological processes.

### 2.10 High-dimensional weighted gene coexpression network analysis

transFusion utilizes high-dimensional weighted gene coexpression network analysis (hdWGCNA) (Morabito *et al.* 2023) to construct a coexpression network based on ST data, using the hdWGCNA package. First, the MetacellsByGroups function is used to generate a metacell gene expression matrix. Then, the TestSoftPowers function is applied to identify the optimal soft power. Finally, the ConstructNetwork function is utilized to construct the coexpression network that captures gene–gene relationships within the spatial context of the tissue.

## 3 Results

**Figure 1:**
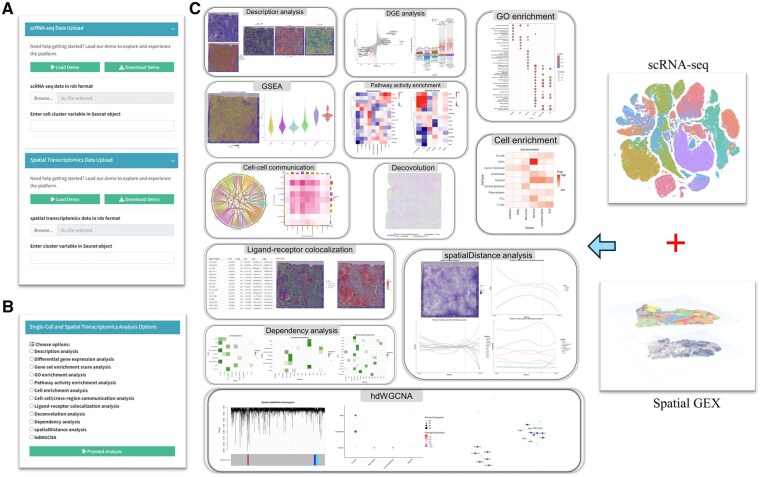
Features of transFusion. (A) Data upload module. (B, C) Analytical modules.

transFusion is a user-friendly and powerful tool developed to streamline the analysis of human and mouse scRNA-seq and 10× Visium ST data, leveraging advanced analytical techniques. The platform consists of two main modules: a data upload module (Fig. 1A) and an analytical module (Fig. 1B and C). The data upload module allows users to input Seurat objects containing scRNA-seq and 10× Visium ST data, while the analytical module includes 12 key functions that enable comprehensive data analysis (Table 2). The details of 12 functions can be found in the “Materials and methods” section. Users can design customized analytical pipelines by selecting from these functions, offering flexibility and adaptability for various research objectives.

**Table 2:** Overview of analytical techniques for spatial and single-cell transcriptomics analysis.

Analysis module	Description
Descriptive analysis	Visualizes gene expression, cell distribution, and pathway activity across spatial regions
DGE analysis	Identifies genes with differential expression across conditions or regions
GSEA	Assesses groups of genes to determine significant enrichment in specific biological processes
GO enrichment analysis	Explores significant biological processes and functional pathways
Pathway activity enrichment analysis	Uncovers important active signaling pathways in distinct regions or clusters
Cell enrichment analysis	Identifies the enrichment of cell types across different regions or clusters
Cell–cell and cross-region communication analysis	Examines interactions between cells or across spatial regions based on ligand–receptor expression
Ligand–receptor colocalization analysis	Detects and visualizes spatially proximate ligand–receptor pairs in ST data
Deconvolution analysis	Resolves mixed cell populations within spots to identify the cell-type composition
Dependency analysis	Studies the spatial dependency of cells or pathways
spatialDistance analysis	Analyzes tissue organization by exploring spatial gradient patterns of cell composition, gene expression, and signaling pathway activity
hdWGCNA	Identifies gene coexpression modules, revealing regulatory networks, and biological function modules

### 3.1 Case study 1: Characterizing cell–cell interactions within the tumor microenvironment of human breast cancer

**Figure 2:**
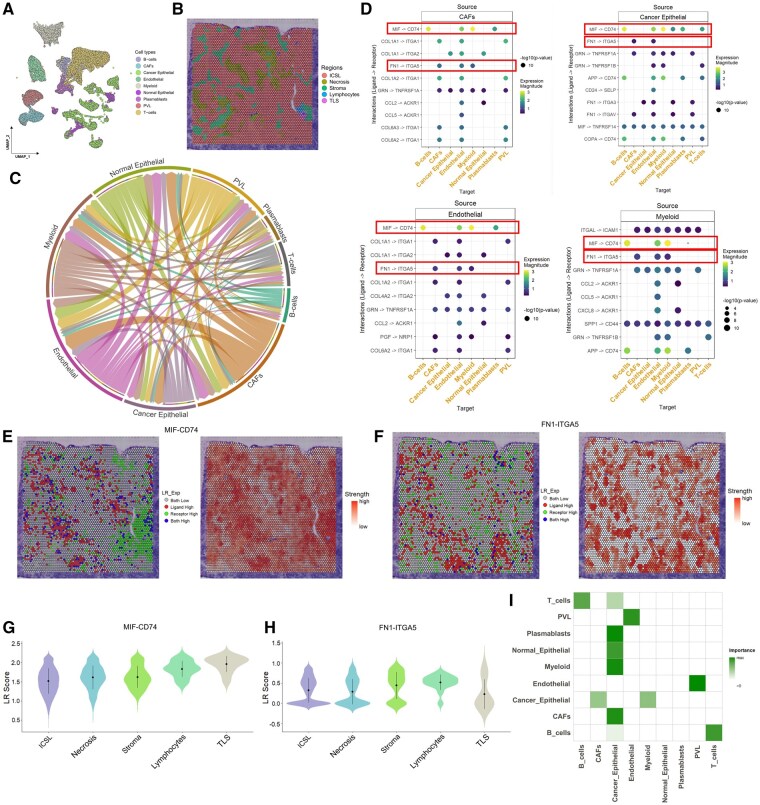
Integrative analysis of scRNA-seq and ST reveals distinct cell–cell interaction patterns and spatial organization in the breast cancer microenvironment. (A) Uniform manifold approximation and projection (UMAP) of breast cancer scRNA-seq data. (B) Spatial transcriptomic spot colored by region annotation. (C), Chord diagram of the intercellular communication network. Edge richness represents the number of ligand–receptor interactions. (D) Dot plots of ligand–receptor interactions between specific cell types. (E, F) Visualization of selected ligand–receptor pairs for ST data. Left is the relative expression of the ligand and the receptor; right is the interaction strength. (G, H) Violin plots of the interaction strength of specified ligand–receptor pairs on different annotated regions. (I) Degree of intercellular dependence within spots. ICSL, invasive cancer + stroma + lymphocytes; PVL, perivascular-like cells; TLS, tertiary lymphoid structures.

The tumor microenvironment (TME) is a complex network of interactions between cancer cells and surrounding components, driving tumor progression, metastasis, and drug resistance (Wang *et al.* 2023). Gaining insight into these cellular interactions within the TME is essential for developing novel therapeutic strategies (Zhang *et al.* 2024). In this study, we applied transFusion to decipher cell–cell interactions within the TME of human breast cancer via integrating scRNA-seq data (Fig. 2A) derived from 21 breast cancer patients (Wu *et al.* 2021) and 10× Visium ST data (Fig. 2B) from a breast cancer patient (Wu *et al.* 2021).

To decipher the complex intercellular communication network within the TME, we first employed CellPhoneDB analysis on the scRNA-seq dataset. Among the nine distinct cell populations identified in the TME, cancer-associated fibroblasts (CAFs), endothelial cells, and myeloid cells demonstrated the most extensive interaction networks with other cell types (Fig. 2C). Further investigation of ligand–receptor pairs revealed significant crosstalk between these three cell populations and cancer epithelial cells and other cells (Fig. 2D). Notably, we identified two predominant signaling axes shared: the MIF-CD74, which has been previously implicated in tumor immune evasion (Wang *et al.* 2017), and FN1-ITGA5, which has been associated with promoting tumor cell viability, invasion, and migration (Cai *et al.* 2018). To understand the spatial distribution patterns of these interactions, we performed ligand–receptor coexpression analysis using ST data. By integrating this with histological annotations (Fig. 2B) from H&E-stained images, compared to tumor invasion areas, MIF-CD74 coexpression levels were higher in noninvasion regions, and FN1-ITGA5 showed stronger enrichment in stromal and lymphocyte areas (Fig. 2E–H). The elevated MIF-CD74 signaling in noninvasion areas may represent an immunosuppressive barrier that precedes tumor invasion, potentially creating a permissive environment for subsequent tumor progression. Meanwhile, the enrichment of FN1-ITGA5 in stromal and lymphocyte regions likely reflected active stromal remodeling and invasion processes, regulated by the crosstalk between CAFs, endothelial cells, and immune cells in these areas. To comprehensively characterize cellular relationships, we applied CARD (Ma and Zhou 2022) to perform deconvolution analysis (Table S1, available as supplementary data at *Bioinformatics* online) and conducted dependency analysis on the ST data to evaluate whether cell-type abundances within spots could be predicted by their neighboring cellular composition. This analysis revealed that myeloid cells, plasmablasts, and T cells were highly predictive of the abundance of cancer epithelial cells within all spots, and CAFs and cancer epithelial cells were colocalized, suggesting potential interdependencies among these TME components (Fig. 2I).

### 3.2 Case study 2: Deciphering the spatial architecture of pancreatic ductal adenocarcinoma

**Figure 3:**
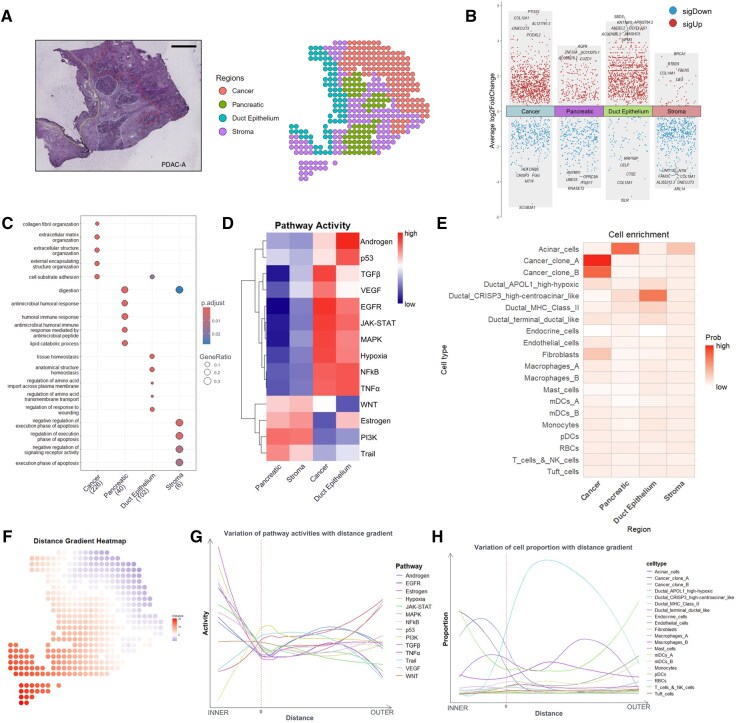
Integrative analysis of scRNA-seq and ST deciphers the spatial architecture of pancreatic ductal adenocarcinoma. (A) H&E-stained image of PDAC (left) and annotated spatial regions of PDAC data (right). (B) Volcano plot of differential gene expression among regions. (C) Comparison of GO enrichment among regions. (D) PROGENy pathway activities across different regions. (E) Cell enrichment analysis of regions. (F) Gradient heatmap of distance from the leading edge of PDAC. (G) Variation of PROGENy pathway activities with distance gradient. (H) Variation of cellular proportion with distance gradient.

The spatial organization of tumor tissue represents a critical determinant in cancer biology, significantly influencing disease progression and therapeutic outcomes (Arora *et al*. 2023). During tumor evolution, cancer cells progressively infiltrate and restructure adjacent tissues, establishing spatially distinct compartments with unique molecular and cellular characteristics. Pancreatic ductal adenocarcinoma (PDAC) is characterized by a prominent stromal microenvironment with significant cellular and spatial heterogeneity that profoundly affects disease biology and treatment resistance (Sherman and Beatty 2023). In this study, we applied transFusion to illustrate the spatial architecture of PDAC by integrating scRNA-seq and ST data generated from the same PDAC patient (Moncada *et al.* 2020). According to histological characteristics, the PDAC tissue was segmented into cancer, pancreatic, ductal, and stromal regions (Fig. 3A). First, DGE analysis was performed to investigate the gene expression profiles characteristic of each region (Table S2, available as supplementary data at *Bioinformatics* online). The result illustrated that the cancer cell regions were primarily enriched with genes related to tumor progression and invasion, like PTGS2 (Lin *et al.* 2019), COL12A1 (Tang *et al.* 2023), and ONECUT3 (Prajapati *et al.* 2024), whereas the stromal regions displayed gene signatures such as BRCA1 (Krishnan *et al.* 2021), BTBD9 (Li *et al.* 2020) suggested early microenvironmental adaptations that may precondition the tissue for tumor development. Differentially expressed genes in the ductal epithelial regions reflected the structural and functional characteristics of epithelial cells, while the pancreatic regions exhibited genes linked to normal physiological functions (Fig. 3B). To further investigate the biological functional significance of the variable genes across distinct regions, we applied Gene Ontology (GO) enrichment analysis and pathway activity enrichment analysis. The results indicated that the cancer cell regions were significantly enriched in pathways associated with extracellular matrix remodeling. In the pancreatic regions, enrichment was observed in immune response, digestion and metabolic processes, consistent with the normal physiological functions of pancreatic tissue. The ductal epithelial regions were enriched in pathways related to tissue homeostasis, amino acid transport, and response to injury. Additionally, the stromal regions showed significant enrichment in apoptosis regulation pathways (Fig. 3C). Results of pathway activity analysis provided an overview of the differential activation of key signaling pathways across different regions of PDAC tissue. We found that in the cancer region, pathways including TGFβ, VEGF, EGFR, JAK-STAT, MAPK, NFkB, TNFα, and Hypoxia show high activity (Fig. 3D).

Building upon our insights into spatial transcriptional signatures, we next sought to deconvolve the cell-type heterogeneity across distinct tissue regions. To this end, we performed cell-type enrichment analysis, which revealed region-specific differences in cellular compositions. From the result, we observed that the cancer region was mainly enriched with cancer clone A, cancer clone B, and fibroblasts, the duct epithelium region was mainly enriched with ductal cells, while the pancreatic and stroma regions were enriched with acinar cells (Fig. 3E). Since swelling and invasive growth are the biological characteristics of tumor cells, to further understand how PDAC invades and remodels its surrounding microenvironment, we performed the CARD (Table S3, available as supplementary data at *Bioinformatics* online) and spatialDistance analysis on ST data to check whether there were gradient-like changes of cellular proportion and pathway activities in the direction perpendicular to the leading edge on both sides of PDAC (Fig. 3F). The results revealed that in the tumor regions, pathway activity demonstrated a clear gradient effect, with pathways such as TGFβ, p53, Androgen, VEGF, EGFR, JAK-STAT, MAPK, NFkB, TNFα, and Hypoxia decreasing as the distance from the tumor edge increased. In contrast, pathways like PI3K, Trail and Estrogen exhibited the opposite trend, showing increased activity further from the tumor boundary (Fig. 3G). For cellular proportion gradient-like changes, we observed that fibroblast proportions were the lowest around the cancer boundary, while ductal crisp3 high-centroacinar-like cells were the highest (Fig. 3H).

## 4 Discussion

Single-cell and spatial transcriptomics have emerged as powerful tools for understanding tissue heterogeneity and cellular interactions within their native context. However, the integrative analysis of scRNA-seq and 10× Visium ST data requires sophisticated computational skills and statistical expertise that often present significant technical barriers for many researchers. In this study, we present transFusion, an integrative platform that addresses a critical gap by advancing the analysis of 10× Visium ST and scRNA-seq data. The platform integrates a comprehensive toolkit, including deconvolution analysis, ligand–receptor colocalization, pathway activity analysis and additional advanced methods, empowering users to decipher complex spatial molecular architectures, regulatory networks, and intercellular interactions within the native tissue microenvironments. These tools enable detailed exploration of intercellular interactions and spatial variation patterns within the TME, as evidenced in breast cancer and PDAC case studies.

In our breast cancer case study, the platform revealed extensive cellular communication networks, particularly highlighting the intricate interactions between CAFs, endothelial cells, and myeloid cells. The identification of two predominant signaling axes, MIF-CD74 and FN1-ITGA5, demonstrated distinct spatial distribution patterns that reflect the dynamic nature of tumor–stromal interactions. Notably, the elevated MIF-CD74 signaling in noninvasion areas and the enrichment of FN1-ITGA5 in stromal and lymphocyte regions suggested regional specialization in immune regulation and angiogenic processes. These spatial patterns offer valuable insights into the mechanisms of tumor progression and potential therapeutic targeting strategies, particularly for disrupting specific tumor–stromal interactions within distinct microenvironmental regions. In the PDAC case study, through the identification of spatial gradients in pathway activities and cellular proportions, transFusion captured molecular changes across tissue boundaries, particularly within the TME. This gradient-based analysis offers a dynamic view of tissue remodeling and reveals key trends in pathways associated with tumor progression and immune evasion. The insights gained here may directly inform therapeutic strategies targeting tumor–stromal interactions and regional cellular heterogeneity.

transFusion offers customizable analytical pipelines, allowing researchers to tailor analyses for a broad spectrum of research goals, from exploratory tissue architecture studies to hypothesis-driven inquiries into specific cellular behaviors. This flexibility is essential given the diverse research questions in spatial biology, spanning fields from developmental biology to oncology. By reducing the statistical and computational barriers of spatial and single-cell data integration, transFusion democratizes advanced analysis methods, making them accessible to a wider scientific audience, including those without specialized computational programming skills and statistical expertise. This ease of use is anticipated to accelerate research progress by enabling more scientists to conduct spatial analyses effectively.

The widespread adoption of 10× Visium technology has generated a wealth of valuable data that still holds significant potential for biological discovery (Yue *et al.* 2023). Furthermore, the relatively lower cost and established protocols of 10× Visium technology enable larger-scale studies across multiple samples and conditions, facilitating robust statistical analyses and broader biological insights. However, higher resolution ST technologies can offer unprecedented detail at subcellular scales. Although this platform can currently be adapted for analysis by merging bins to lower sequencing resolution, expanding compatibility to high-resolution ST datasets, such as Visium HD and Stereo-seq, will further bridge the gap between spot-based and single-cell analyses in the future. Future developments of transFusion will focus on expanding its analytical capabilities on higher resolution ST technologies and across multiple dimensions by integrating emerging analytical methods. This enhancement will collectively strengthen transFusion’s utility as a comprehensive platform for ST analysis, advancing our understanding of complex tissue microenvironments across various biological contexts and experimental scales.

In summary, transFusion not only addresses current limitations in spatial and single-cell data analysis but also elevates the level of insight that can be obtained from these technologies. Its applications are broad, spanning multiple research domains and enhancing the depth of understanding of tissue organization and function. It represents a pivotal advancement in spatial biology, with the potential to drive the field toward a more comprehensive understanding of tissue structure and function.

## Supplementary Material

btag059_Supplementary_Data

## Data Availability

For case study 1, the scRNA-seq data of human breast cancer are available through the Gene Expression Omnibus (GEO) under accession number GSE176078. ST data of human breast cancer is available from the Zenodo data repository (DOI: 10.5281/zenodo.4739739). For case study 2, the human PDAC dataset is available through the GEO under accession number GSE111672. transFusion is freely available in GitHub at https://github.com/WQLin8/transFusion and the source code has been archived on Zenodo (DOI: 10.5281/zenodo.18200659).

## References

[btag059-B1] Andersson A , Bergenstråhle J, Asp M et al Single-cell and spatial transcriptomics enables probabilistic inference of cell type topography. Commun Biol 2020;3:565. 33037292 10.1038/s42003-020-01247-y PMC7547664

[btag059-B2] Arora R , Cao C, Kumar M et al Spatial transcriptomics reveals distinct and conserved tumor core and edge architectures that predict survival and targeted therapy response. Nat Commun 2023;14:14. 10.1038/s41467-023-40271-4 37596273 PMC10439131

[btag059-B3] Badia-I-Mompel P , Vélez Santiago J, Braunger J et al decoupleR: ensemble of computational methods to infer biological activities from omics data. Bioinform Adv 2022;2:vbac016. 36699385 10.1093/bioadv/vbac016 PMC9710656

[btag059-B4] Birnbaum KD. Power in numbers: single-cell RNA-seq strategies to dissect complex tissues. Annu Rev Genet 2018;52:203–21. 30192636 10.1146/annurev-genet-120417-031247 PMC6314027

[btag059-B5] Cable DM , Murray E, Zou LS et al Robust decomposition of cell type mixtures in spatial transcriptomics. Nat Biotechnol 2022;40:517–26. 33603203 10.1038/s41587-021-00830-w PMC8606190

[btag059-B6] Cai X , Liu C, Zhang T-N et al Down‐regulation of FN1 inhibits colorectal carcinogenesis by suppressing proliferation, migration, and invasion. J Cell Biochem 2018;119:4717–28. 29274284 10.1002/jcb.26651

[btag059-B7] Chen A , Liao S, Cheng M et al Spatiotemporal transcriptomic atlas of mouse organogenesis using DNA nanoball-patterned arrays. Cell 2022;185:1777–92.e21. 35512705 10.1016/j.cell.2022.04.003

[btag059-B8] Dong M , Thennavan A, Urrutia E et al SCDC: bulk gene expression deconvolution by multiple single-cell RNA sequencing references. Brief Bioinform 2021;22:416–27. 31925417 10.1093/bib/bbz166 PMC7820884

[btag059-B9] Dong R , Yuan G-C. SpatialDWLS: accurate deconvolution of spatial transcriptomic data. Genome Biol 2021;22:145. 33971932 10.1186/s13059-021-02362-7 PMC8108367

[btag059-B10] Efremova M , Vento-Tormo M, Teichmann SA et al CellPhoneDB: inferring cell–cell communication from combined expression of multi-subunit ligand–receptor complexes. Nat Protoc 2020;15:1484–506. 32103204 10.1038/s41596-020-0292-x

[btag059-B11] Elosua-Bayes M , Nieto P, Mereu E et al SPOTlight: seeded NMF regression to deconvolute spatial transcriptomics spots with single-cell transcriptomes. Nucleic Acids Res 2021;49:e50. 33544846 10.1093/nar/gkab043 PMC8136778

[btag059-B12] Fang S , Chen B, Zhang Y et al Computational approaches and challenges in spatial transcriptomics. Genomics Proteomics Bioinformatics 2023;21:24–47. 36252814 10.1016/j.gpb.2022.10.001 PMC10372921

[btag059-B13] Gong T , Szustakowski JD. DeconRNASeq: a statistical framework for deconvolution of heterogeneous tissue samples based on mRNA-Seq data. Bioinformatics 2013;29:1083–5. 23428642 10.1093/bioinformatics/btt090

[btag059-B14] Hasanaj E , Wang J, Sarathi A et al Interactive single-cell data analysis using Cellar. Nat Commun 2022;13:1998. 35422041 10.1038/s41467-022-29744-0 PMC9010407

[btag059-B15] Holland CH , Szalai B, Saez-Rodriguez J. Transfer of regulatory knowledge from human to mouse for functional genomics analysis. Biochim Biophys Acta Gene Regul Mech 2020;1863:194431. 31525460 10.1016/j.bbagrm.2019.194431

[btag059-B16] Jin S , Plikus MV, Nie Q. CellChat for systematic analysis of cell–cell communication from single-cell transcriptomics. Nat Protoc 2025;20:180–219. 39289562 10.1038/s41596-024-01045-4

[btag059-B17] Kleshchevnikov V , Shmatko A, Dann E et al Cell2location maps fine-grained cell types in spatial transcriptomics. Nat Biotechnol 2022;40:661–71. 35027729 10.1038/s41587-021-01139-4

[btag059-B18] Krishnan R , Patel PS, Hakem R. BRCA1 and metastasis: outcome of defective DNA repair. Cancers (Basel) 2021;14:108. 35008272 10.3390/cancers14010108 PMC8749860

[btag059-B19] Lähnemann D , Köster J, Szczurek E et al Eleven grand challenges in single-cell data science. Genome Biol 2020;21:31–5. 32033589 10.1186/s13059-020-1926-6 PMC7007675

[btag059-B20] Li L , Zhang W, Liu Y et al The CRL3BTBD9 E3 ubiquitin ligase complex targets TNFAIP1 for degradation to suppress cancer cell migration. Signal Transduct Target Ther 2020;5:42. 32327643 10.1038/s41392-020-0140-z PMC7181851

[btag059-B21] Li X , Xiao C, Qi J et al STellaris: a web server for accurate spatial mapping of single cells based on spatial transcriptomics data. Nucleic Acids Res 2023;51:W560–8. 37224539 10.1093/nar/gkad419 PMC10320151

[btag059-B22] Lin W , Li Y, Qiu C et al Mapping the spatial atlas of the human bone tissue integrating spatial and single-cell transcriptomics. Nucleic Acids Res 2025;53:gkae1298. 39817519 10.1093/nar/gkae1298 PMC11736439

[btag059-B23] Lin X-M , Luo W, Wang H et al The role of prostaglandin-endoperoxide synthase-2 in chemoresistance of non-small cell lung cancer. Front Pharmacol 2019;10:836. 31440159 10.3389/fphar.2019.00836 PMC6694719

[btag059-B24] Liu Q , Hsu C-Y, Shyr Y. Scalable and model-free detection of spatial patterns and colocalization. Genome Res 2022;32:1736–45. 36223499 10.1101/gr.276851.122 PMC9528978

[btag059-B25] Ma Y , Zhou X. Spatially informed cell-type deconvolution for spatial transcriptomics. Nat Biotechnol 2022;40:1349–59. 35501392 10.1038/s41587-022-01273-7 PMC9464662

[btag059-B26] Miller BF , Huang F, Atta L et al Reference-free cell type deconvolution of multi-cellular pixel-resolution spatially resolved transcriptomics data. Nat Commun 2022;13:2339. 35487922 10.1038/s41467-022-30033-z PMC9055051

[btag059-B27] Moncada R , Barkley D, Wagner F et al Integrating microarray-based spatial transcriptomics and single-cell RNA-seq reveals tissue architecture in pancreatic ductal adenocarcinomas. Nat Biotechnol 2020;38:333–42. 31932730 10.1038/s41587-019-0392-8

[btag059-B28] Morabito S , Reese F, Rahimzadeh N et al hdWGCNA identifies co-expression networks in high-dimensional transcriptomics data. Cell Rep Methods 2023;3:100498. 37426759 10.1016/j.crmeth.2023.100498 PMC10326379

[btag059-B29] Ospina OE , Manjarres-Betancur R, Gonzalez-Calderon G et al spatialGE is a user-friendly web application that facilitates spatial transcriptomics data analysis. Cancer Res 2025;85:848–58. 39636739 10.1158/0008-5472.CAN-24-2346 PMC11873723

[btag059-B30] Prajapati KS et al Role of ONECUT family transcription factors in cancer and other diseases. Exp Cell Res 2024:114035. 38593917 10.1016/j.yexcr.2024.114035

[btag059-B31] Regev A , Teichmann SA, Lander ES, et al Human Cell Atlas Meeting Participants. The human cell atlas. elife 2017;6:e27041. 29206104 10.7554/eLife.27041 PMC5762154

[btag059-B32] Robles-Remacho A , Sanchez-Martin RM, Diaz-Mochon JJ. Spatial transcriptomics: emerging technologies in tissue gene expression profiling. Anal Chem 2023;95:15450–60. 37814884 10.1021/acs.analchem.3c02029 PMC10603609

[btag059-B33] Rodriques SG , Stickels RR, Goeva A et al Slide-seq: a scalable technology for measuring genome-wide expression at high spatial resolution. Science 2019;363:1463–7. 30923225 10.1126/science.aaw1219 PMC6927209

[btag059-B34] Schubert M , Klinger B, Klünemann M et al Perturbation-response genes reveal signaling footprints in cancer gene expression. Nat Commun 2018;9:20. 29295995 10.1038/s41467-017-02391-6 PMC5750219

[btag059-B35] Sherman MH , Beatty GL. Tumor microenvironment in pancreatic cancer pathogenesis and therapeutic resistance. Annu Rev Pathol 2023;18:123–48. 36130070 10.1146/annurev-pathmechdis-031621-024600 PMC9877114

[btag059-B36] Shi J , Wei X, Xun Z et al The web-based portal SpatialTME integrates histological images with single-cell and spatial transcriptomics to explore the tumor microenvironment. Cancer Res 2024;84:1210–20. 38315776 10.1158/0008-5472.CAN-23-2650

[btag059-B37] Ståhl PL , Salmén F, Vickovic S et al Visualization and analysis of gene expression in tissue sections by spatial transcriptomics. Science 2016;353:78–82. 27365449 10.1126/science.aaf2403

[btag059-B38] Stuart T , Butler A, Hoffman P et al Comprehensive integration of single-cell data. Cell 2019;177:1888–902.e21. 31178118 10.1016/j.cell.2019.05.031 PMC6687398

[btag059-B39] Stubbington MJT , Rozenblatt-Rosen O, Regev A et al Single-cell transcriptomics to explore the immune system in health and disease. Science 2017;358:58–63. 28983043 10.1126/science.aan6828 PMC5654495

[btag059-B40] Tanevski J , Flores ROR, Gabor A et al Explainable multiview framework for dissecting spatial relationships from highly multiplexed data. Genome Biol 2022;23:97. 35422018 10.1186/s13059-022-02663-5 PMC9011939

[btag059-B41] Tang Z , Yang Y, Zhang Q et al Epigenetic dysregulation-mediated COL12A1 upregulation predicts worse outcome in intrahepatic cholangiocarcinoma patients. Clin Epigenetics 2023;15:13. 36694230 10.1186/s13148-022-01413-5 PMC9875497

[btag059-B42] Tokura M , Nakayama J, Prieto-Vila M et al Single-cell transcriptome profiling reveals intratumoral heterogeneity and molecular features of ductal carcinoma in situ. Cancer Res 2022;82:3236–48. 35852797 10.1158/0008-5472.CAN-22-0090

[btag059-B43] Tsoucas D , Dong R, Chen H et al Accurate estimation of cell-type composition from gene expression data. Nat Commun 2019;10:2975. 31278265 10.1038/s41467-019-10802-z PMC6611906

[btag059-B44] Wang Q , Shao X, Zhang Y et al Role of tumor microenvironment in cancer progression and therapeutic strategy. Cancer Med 2023;12:11149–65. 36807772 10.1002/cam4.5698 PMC10242329

[btag059-B45] Wang X , Park J, Susztak K et al Bulk tissue cell type deconvolution with multi-subject single-cell expression reference. Nat Commun 2019;10:380. 30670690 10.1038/s41467-018-08023-x PMC6342984

[btag059-B46] Wang Y , Luo J, Jiao S, et al STExplore: an integrated online platform for comprehensive analysis and visualization of spatial transcriptomics data. Small Methods 2025;9:e2401272. 40045664 10.1002/smtd.202401272

[btag059-B47] Wang Z-Q , Milne K, Webb JR et al CD74 and intratumoral immune response in breast cancer. Oncotarget 2017;8:12664–74. 27058619 10.18632/oncotarget.8610 PMC5355043

[btag059-B48] Wu SZ , Al-Eryani G, Roden DL et al A single-cell and spatially resolved atlas of human breast cancers. Nat Genet 2021;53:1334–47. 34493872 10.1038/s41588-021-00911-1 PMC9044823

[btag059-B49] Wu T , Hu E, Xu S et al clusterProfiler 4.0: a universal enrichment tool for interpreting omics data. Innovation 2021;2:100141. 34557778 10.1016/j.xinn.2021.100141 PMC8454663

[btag059-B50] Yang S , Zhou X. SRT-Server: powering the analysis of spatial transcriptomic data. Genome Med 2024;16:18. 38279156 10.1186/s13073-024-01288-6 PMC10811909

[btag059-B51] Yue L , Liu F, Hu J et al A guidebook of spatial transcriptomic technologies, data resources and analysis approaches. Comput Struct Biotechnol J 2023;21:940–55. 38213887 10.1016/j.csbj.2023.01.016 PMC10781722

[btag059-B52] Zhang L et al Targets of tumor microenvironment for potential drug development. MedComm Oncol 2024;3:e68.

[btag059-B53] Zhang Y , Li T, Wang G et al Advancements in single-cell RNA sequencing and spatial transcriptomics for central nervous system disease. Cell Mol Neurobiol 2024;44:65. 39387975 10.1007/s10571-024-01499-w PMC11467076

[btag059-B54] Zhang Z , Cui F, Su W et al webSCST: an interactive web application for single-cell RNA-sequencing data and spatial transcriptomic data integration. Bioinformatics 2022;38:3488–9. 35604082 10.1093/bioinformatics/btac350

[btag059-B55] Zheng Y , Chen Y, Ding X et al Aquila: a spatial omics database and analysis platform. Nucleic Acids Res 2023;51:D827–34. 36243967 10.1093/nar/gkac874 PMC9825501

